# Esophageal Neuroendocrine Carcinoma Presenting After Definitive Chemoradiation of Squamous Cell Carcinoma in the Same Location

**DOI:** 10.14309/crj.0000000000001091

**Published:** 2023-06-19

**Authors:** Zarian Prenatt, Hammad Liaquat, Brittney Shupp, Lisa Stoll, Yecheskel Schneider

**Affiliations:** 1 Department of Internal Medicine, St. Luke's University Health Network, Bethlehem, PA; 2 Department of Gastroenterology, St. Luke's University Health Network, Bethlehem, PA; 3 Department of Pathology, St. Luke's University Health Network, Bethlehem, PA

**Keywords:** esophagus, esophageal neuroendocrine carcinoma, squamous cell carcinoma, radiation-induced malignancy, chemoradiation

## Abstract

Esophageal neuroendocrine carcinoma is very rare and highly aggressive. An 85-year-old man with a history of esophageal squamous cell carcinoma in remission presented 4 years after definitive chemoradiation with new-onset dysphagia. Endoscopy with biopsy revealed high-grade malignancy consistent with neuroendocrine carcinoma. Treatment options were limited to chemotherapy because of his metastatic disease, and he unfortunately died 14 months after diagnosis. The occurrence of esophageal neuroendocrine carcinoma in a site of prior squamous cell carcinoma is very uncommon, and this likely represents a case of radiation-induced malignancy. Therefore, when undergoing radiotherapy, patients and providers should discuss the possibility of this life-threatening complication.

## INTRODUCTION

Esophageal neuroendocrine carcinoma (ENEC) is an extremely rare and highly aggressive malignancy that carries a poor prognosis.^1^ The incidence of ENEC between 2000 and 2016 was reported to be low at approximately 0.044 per 100,000 persons, and studies have demonstrated a 5-year survival rate of 4.8%.^1,2^ Over 50% of patients with cancer receive radiotherapy as part of their treatment regimen.^3^ Second cancer development is an established risk of radiotherapy, and neuroendocrine carcinomas have been shown to arise from irradiated locations.^4^ This report describes a rare case of ENEC presenting several years after chemoradiation of esophageal squamous cell carcinoma (SCC) in the same location.

## CASE REPORT

**Figure 1.**
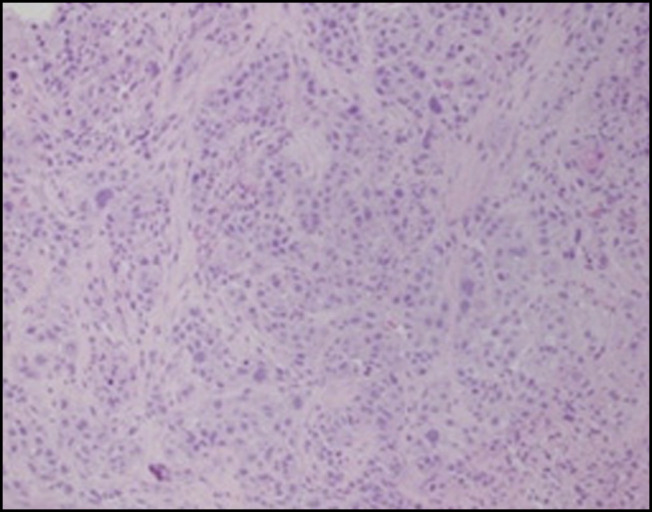
Hematoxylin and eosin stain showing moderately differentiated squamous cell carcinoma of the esophagus (20× magnification).

An 85-year-old White man presented for oncology follow-up with 2 months of progressive dysphagia to solids and no additional associated symptoms. The patient had no history of tobacco or alcohol use, but his medical history was significant for T4N0 grade I SCC of the upper esophagus diagnosed 5 years prior (Figure 1). He previously received chemoradiation with 4-year remission confirmed by annual computed tomography (CT) and upper endoscopy; however, routine biopsies of the prior SCC site were not performed during endoscopic surveillance. The previous total radiation dose received was 50.4 gray (Gy), and he was treated with paclitaxel and carboplatin for 5 weeks.

**Figure 2.**
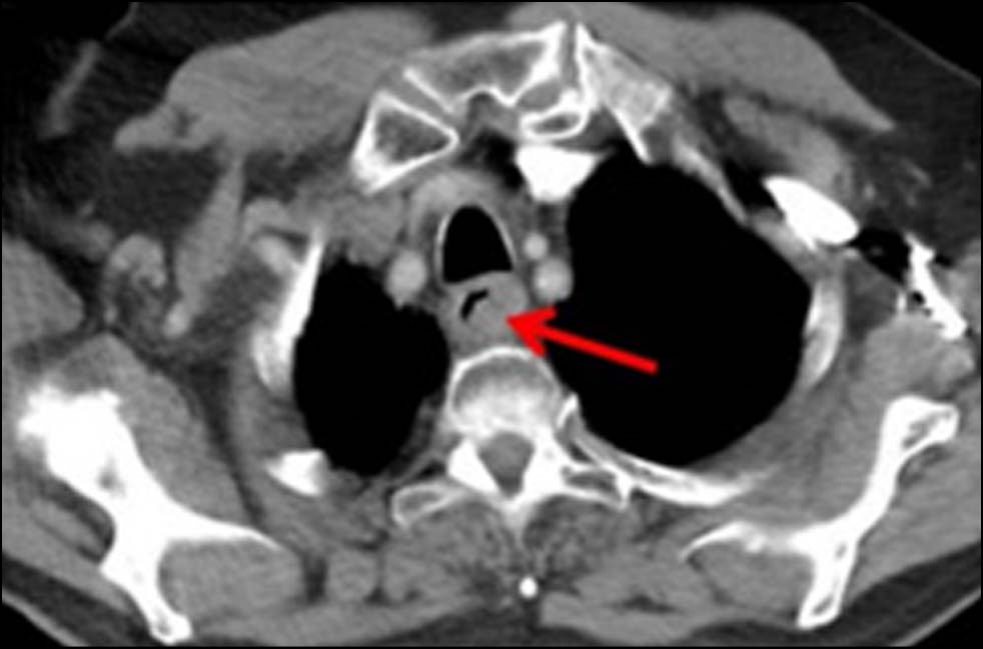
Chest CT showing soft-tissue thickening (red arrow) in the proximal third of the esophagus compatible with recurrent tumor. CT, computed tomography.

**Figure 3.**
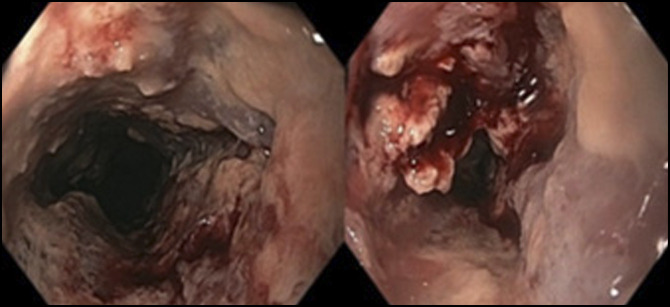
EGD showing friable, hemorrhagic, and ulcerated mucosa in the upper third of the esophagus. EGD, esophagogastroduodenoscopy.

**Figure 4.**
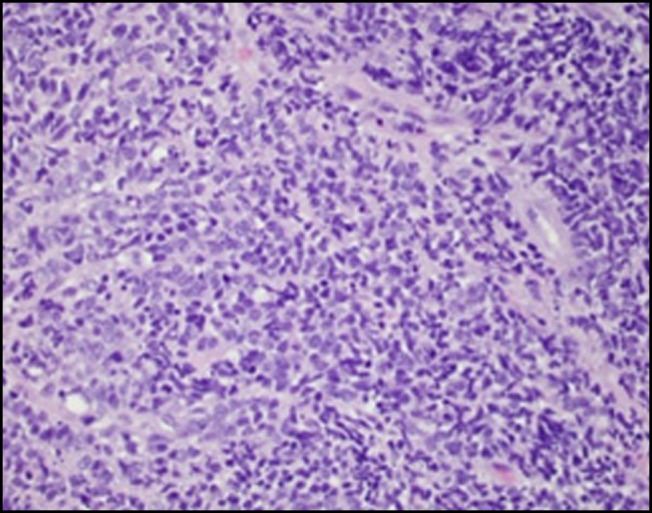
Hematoxylin and eosin stain of the esophagus showing sheets of neuroendocrine carcinoma with small blue cells containing scant cytoplasm and nuclear molding (40× magnification).

**Figure 5.**
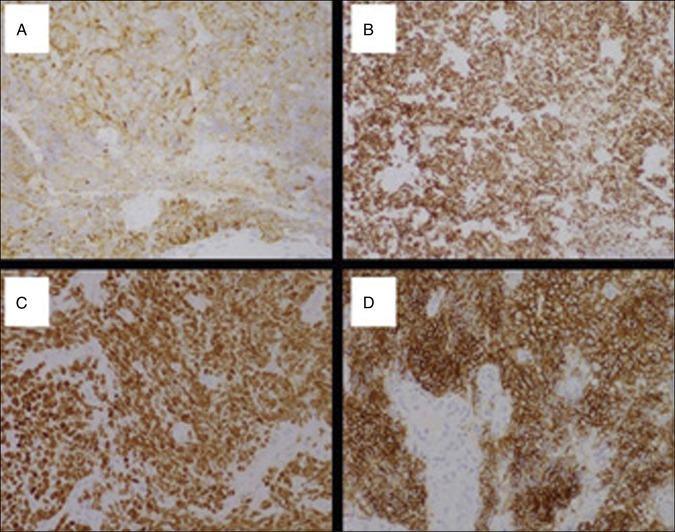
(A) Synaptophysin immunohistochemical stain showing diffuse cytoplasmic staining confirming neuroendocrine nature of the tumor (40× magnification). (B) Cytokeratin AE1/AE3 (CK AE 1/3) immunohistochemistry showing membranous staining confirming carcinoma (10× magnification). (C) Thyroid transcription factor 1 immunostaining confirming small cell carcinoma (20× magnification). (D) CD56 immunostaining supporting the diagnosis of neuroendocrine carcinoma (20× magnification).

**Figure 6.**
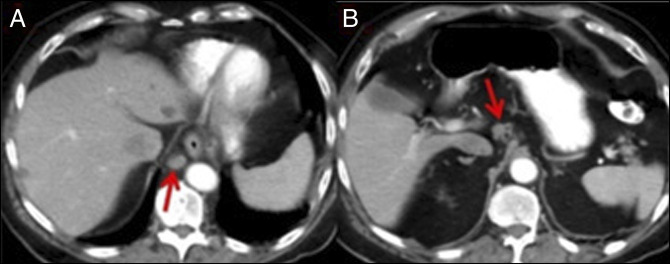
(A) CT showing a newly enlarged right retrocrural lymph node (red arrow) measuring 1.3 cm in the short axis. (B) CT showing newly enlarged celiac axis lymph nodes (red arrow) measuring 1.2 cm in the short axis. CT, computed tomography.

Given his dysphagia, neck and chest CT with contrast was completed and showed soft-tissue thickening in the upper esophagus at the location of previously treated SCC (Figure 2). An esophagogastroduodenoscopy showed friable and ulcerated mucosa in the upper esophagus (Figure 3), with biopsies revealing high-grade malignancy consistent with poorly differentiated small cell neuroendocrine carcinoma and no evidence of SCC (Figure 4). Immunohistochemistry staining was positive for synaptophysin, cytokeratin AE1/AE3, thyroid transcription factor 1, and CD56 (Figure 5). The Ki-67 mitotic proliferation index was 80%–90%. Abdominal CT also showed newly enlarged abdominal, retroperitoneal, and retrocrural lymph nodes measuring up to 1.3 cm concerning for progressive malignancy (Figure 6).

Given the concern for metastatic disease, medical therapy was pursued instead of surgery. He was not a candidate for radiation because of previous radiotherapy to the same location. The patient underwent chemotherapy with etoposide and carboplatin, but he had disease progression evident on bimonthly CT imaging 6 months after starting treatment. His treatment was briefly switched to irinotecan with subsequent treatment-induced diarrhea. His dysphagia worsened, and he required percutaneous endoscopic gastrostomy tube placement. However, his treatment was stopped, and palliative care was initiated because of the lack of treatment response and worsening performance status. Metastatic lesions in the scalp were discovered on head CT, which were not evident on head CT 4 months earlier. The patient died in hospice care approximately 14 months after the diagnosis of ENEC.

## DISCUSSION

The occurrence of ENEC in a site of prior esophageal SCC is a rarity. This likely represents an example of malignant evolution from radiation-induced injury. Radiotherapy is used to decrease cancer recurrence and improve survival. However, with improved survival, it is important to consider the long-term risks of radiation. Several studies have shown that radiotherapy increases the risk of second cancer development.^5,6^ Berrington de Gonzalez conducted a systemic analysis of 647,672 cancer survivors with an average 12-year follow-up. They found that 60,271 (9%) of those patients developed a second solid cancer, and their data suggest that approximately 8% of second solid cancers may be related to the initial radiation treatment.^5^

Radiation-induced malignancy (RIM) is a delayed life-threatening complication of radiotherapy.^7^ The pathogenesis is thought to be due to DNA breaks with alteration in the DNA repair protein caused by ionizing radiation.^3^ According to the Cahan criteria, for a cancer to be considered radiation induced, the tumor must (i) occur within the previous radiation field, (ii) have temporal relation to the previous radiation with latency of at least 4 years, and (iii) have a different histology from the initial tumor.^8^ Our case likely demonstrates a case of RIM because it fulfills the above criteria. One study evaluated the risk of esophageal RIM in patients who received radiation for breast cancer and found the risk to increase by approximately 0.5% per Gy of radiation.^9^ A radiation dose of 50 Gy, as in our patient, would therefore be associated with an approximate 25% increased risk of developing esophageal cancer. Another study estimating the risk of esophageal RIM in patients who received radiotherapy for Hodgkin lymphoma found the risk to increase by approximately 7% per Gy.^10^ In this case, a dose of 50 Gy would be associated with a 3.5 times higher risk of developing esophageal cancer.

The low incidence of ENEC may be attributed to the underdevelopment of the neuroendocrine system within the esophagus.^1^ ENEC is usually located in the mid to distal esophagus, which is thought to be due to the predominance of endocrine cells within the distal cardiac glands and Merkel cells in the middle esophagus.^11^ Numerous case reports demonstrate that most neuroendocrine carcinomas develop in the distal esophagus and are associated with columnar lining, as seen in Barrett's esophagus.^12^ These findings support the conclusion that an iatrogenic cause contributed to the development of NEC in our patient's upper esophagus.

Given the highly aggressive nature of ENEC, it is important to deliver timely treatments at an early stage.^1^ Unfortunately, treatment approaches are not well defined because of the small number of ENEC cases in the literature.^13^ Current strategies are based on protocols developed for treating NECs at other locations.^13^ Although surgery is the preferred treatment, studies indicate that a significantly lower proportion of patients undergo resection compared with receiving chemoradiation.^13^ This is likely related to the aggressive nature of ENEC because metastases are often present at diagnosis, as in this case.^14^

Patients and providers should discuss the risk of radiation-related cancer, and patients who receive radiation should be followed closely for RIM. The literature on ENEC outcomes and the development of ENEC in a previously irradiated field is scarce, and our case adds to the limited information on this rare phenomenon. Extensive research is required to determine standardized treatment strategies for both primary and radiation-induced ENEC.

## DISCLOSURES

Author contributions: Z. Prenatt wrote the manuscript and reviewed the literature. B. Shupp edited the manuscript. L. Stoll provided the pathology images. H. Liaquat and Y. Schneider revised the manuscript for intellectual content and approved the final manuscript. Z. Prenatt is the article guarantor.

Financial disclosure: None to report.

Previous presentation: This case report was presented at the American College of Gastroenterology Annual Scientific Meeting in Charlotte, NC, on October 25, 2022.

Informed consent was obtained for this case report.
